# Primary pulmonary Hodgkin lymphoma presenting as cavitary lung lesions

**DOI:** 10.36416/1806-3756/e20240338

**Published:** 2024-12-17

**Authors:** Roberta Wartchow Machado, Felipe Welter Langer, Rodrigo dos Santos Ferrari

**Affiliations:** 1. Departamento de Radiologia e Diagnóstico por Imagem, Hospital Universitário de Santa Maria, Santa Maria (RS) Brasil.; 2. Universidade Federal Fronteira Sul, Passo Fundo (RS) Brasil.; 3. Clínica Kozma, Passo Fundo (RS) Brasil.

**Figure 1:**
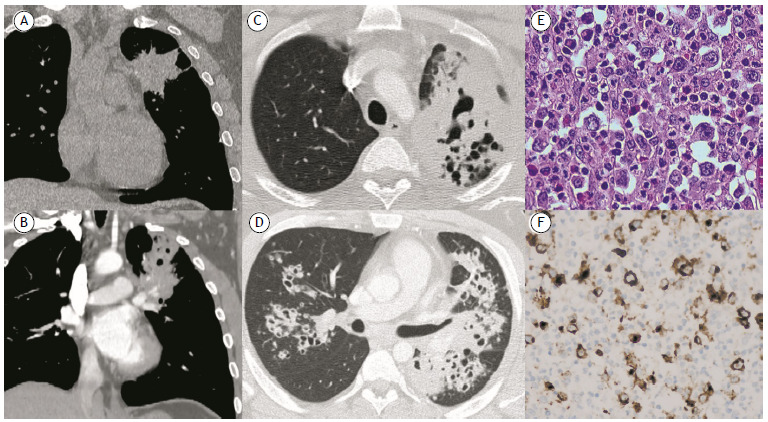
In A, unenhanced chest CT scan showing a mass of 7 cm in width in the left upper lobe. In B, contrast-enhanced chest CT scan performed 70 days after the initial scan, showing an increase in lesion size, as well as central cavitation. In C and D, follow-up contrast-enhanced chest CT scan performed six months after the initial scan, showing new bilateral cavitary peribronchial nodules. In E, histopathological analysis of the excised left upper lobe, showing Reed-Sternberg cells (H&E; magnification, ×20). In F, immunohistochemistry showing positivity for CD30.

A 27-year-old male smoker presented with a six-month history of chest discomfort and hemoptysis. His past medical history was otherwise unremarkable. Tuberculosis and immunodeficiency screenings were negative. An unenhanced chest CT scan revealed a 7-cm mass in the left upper lobe and prevascular lymphadenopathy (Figure 1A). Percutaneous and transbronchial biopsies were inconclusive. Follow-up imaging evidenced an increase in lesion size and central cavitation, as well as new bilateral peribronchial cavitary nodules (Figures 1B-1D). A left upper lobectomy was performed, and histopathological examination of the surgical specimen confirmed the diagnosis of nodular sclerosis Hodgkin lymphoma (Figures 1E and 1F). 

Primary pulmonary Hodgkin lymphoma (PPHL) accounts for less than 1% of all lymphomas, nodular sclerosis being the most common type.^1^ ^,^ ^2^ Symptoms are nonspecific and may include weight loss, fever, dry cough, and chest discomfort. On imaging, PPHL has a predilection for the upper lobes and may present as unilateral or bilateral parenchymal consolidations or nodules, which may cavitate in about 20% of cases.^1^ ^-^ ^3^ Histological confirmation through incisional biopsy may be challenging because of background inflammation and necrosis, excisional biopsy being frequently required to establish a diagnosis.^3^ Therefore, PPHL should be included in the differential diagnosis of otherwise unexplained parenchymal consolidations and cavitary nodules. 
